# Bubbles spray aerosols: Certitudes and mysteries

**DOI:** 10.1093/pnasnexus/pgac261

**Published:** 2022-11-26

**Authors:** Emmanuel Villermaux, Xiaofei Wang, Luc Deike

**Affiliations:** Aix Marseille Université, CNRS, Centrale Marseille, IRPHE UMR 7342, 13384 Marseille, France; Institut Universitaire de France, Paris 75005, France; Department of Environmental Science and Engineering, Shanghai Key Laboratory of Atmospheric Particle Pollution and Prevention, Fudan University, Shanghai 200433, China; Department of Mechanical and Aerospace Engineering, Princeton University, Princeton, NJ 08544, USA; High Meadows Environmental Institute, Princeton University, Princeton, NJ 08544, USA

**Keywords:** aerosols, sprays, bubble bursting, air-sea interactions

## Abstract

Ocean spray aerosol formed by bubble bursting are at the core of a broad range of atmospheric processes: they are efficient cloud condensation nuclei and carry a variety of chemical, biological, and biomass material from the surface of the ocean to the atmosphere. The origin and composition of these aerosols is sensibly controlled by the detailed fluid mechanics of bubble bursting. This perspective summarizes our present-day knowledge on how bursting bubbles at the surface of a liquid pool contribute to its fragmentation, namely to the formation of droplets stripped from the pool, and associated mechanisms. In particular, we describe bounds and yields for each distinct mechanism, and the way they are sensitive to the bubble production and environmental conditions. We also underline the consequences of each mechanism on some of the many air-sea interactions phenomena identified to date. Attention is specifically payed at delimiting the known from the unknown and the certitudes from the speculations.

Significance Statement From the bubbles production mechanisms by wave breaking to their bursting modes once they have reached the surface, we review each mechanism quantitatively and critically, including recently discovered ones, aiming at contrasting their relative importance in the overall aerosols production flux from the sea, and other sources involving bubbles. We advocate that, because air-sea exchanges are ruled by many intermingled, coupled phenomena and are mediated by very sensitive objects—bubbles—precise experiments considering each effect in isolation are needed to reliably predict sea spray emissions from first principles.

## Perspective and facts

Trillions of bubbles burst every second at the surface of the ocean. The fate of these tiny, fragile objects is to ensure equilibria of water vapor and heat with the atmosphere, as well as to disseminate teratons of salt, organic species, bacteria, viruses, and floating particules like microplasctics in the environment every year (6–8).

As a major source of atmospheric aerosols, sea spray affects radiative forcing, cloud formation, precipitations, biomass, and dissolved chemicals distillation then dissemination, as well as human health so that the whole planet equilibrium relies on bursting bubbles mediated aerosols (9). Depending on their radius, bubbles burst according to several distinct liquid fragmentation processes, each producing droplets broadly distributed in size. However, not all droplets sizes generated by each distinct mechanism contribute to all kinds of ocean-atmosphere interactions. We argue that a mechanistic understanding of bursting processes is essential in order to better constrain, and eventually model, the production of sea spray aerosols of various compositions (sea salt and organic), and assess their importance on chemical processes in the atmosphere.

From the bubbles production mechanisms by wave breaking to their bursting modes once they have reached the surface, we review each mechanism quantitatively and critically, including recently discovered ones (4,10), aiming at contrasting their relative importance in the overall aerosols production flux from the sea, and other sources involving bubbles. Part of the difficulties, and inherent interest of the problem, arises from the broad range of length scales involved in the geometry of a surface bubble, and among the bubbles natural assembly.

This perspective reviews our understanding of the physical processes at play in sea spray aerosol production. It is organized as follows: we first give an historical perspective in 1, underlying existing paradoxes. Then we explain the nature of the distinct bursting bubbles mechanisms in 2 and how different bubble sizes produce different types of aerosols. In 3, we comment on the different scenarii accounting for the bubble sizes distributions formed in the upper ocean by breaking waves, and of those actually reaching the surface to burst, as well as the role of various physico-chemical variables on the different processes. Finally, in 4, we discuss the implications of the various mechanisms on the chemical/biological aerosols enrichment processes, concluding that future studies should combine fluid-mechanical and chemical characterizations of aerosols.

Although a bubble is one of the most common objects in our environment, the details of its origins, its life, and the products of its disappearance still hold lots of uncertainties and mysteries.

### Film and jet drops

Since the seminal, thorough, and extremely smart experiments of D. Blanchard, motivated by the depiction of ocean-atmosphere interactions, it has been customary to classify the droplet emission from bursting bubbles into two main sources (this classification originates from the group of N. Dombrowski at Imperial College (12); see also the visionary comments by Mason (5)), as

1. Film drops, resulting from the breakup of the bubble cap.
2. Jet drops, formed by the capillary fission of the jet emitted by the bubble cavity collapse.

Blanchard’s 1963 review (13) gives a useful historical introduction, fundamental motivations, as well as an impressive account of the breath and depth of his own (still ongoing at the time) contributions to the subject.

It is known that the two above mechanisms do not operate on the same ranges of bubble sizes and that they do not produce the same level of fragmentation, regarding both the size and number of the droplets produced.

**Fig. 1.**
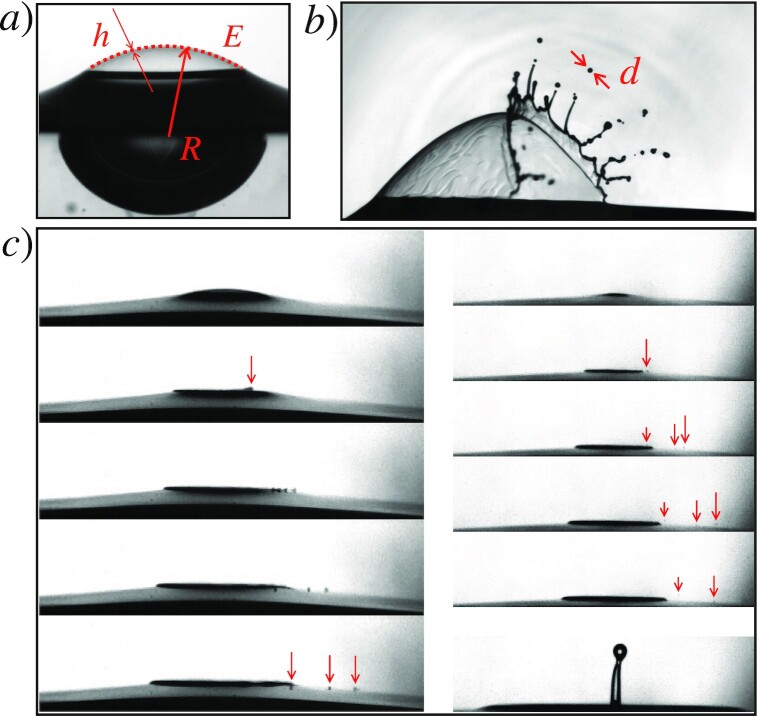
(A) Definition of some geometrical quantities involved in bubble breakup: The radius of curvature of its cap *R* (twice the radius of the immersed part of the bubble, see (1)), the cap extent *E* ∼ *R*^2^/*a* (holding for *R* < *a*, see (2)), the cap thickness $h=R^2/\cal {L}$ with ${\cal L}=\beta a$ and β ≈ 10^3^, see (2,3) while *a* is given in Eq. (1). (B) Regular film drops from a *R* ≳ *a* bubble (*R* ≈ 5 mm), and definition of the droplet size *d*. (C) Film flapping fine droplets formed in SF_6_ (left) and air (right) from *R* ≈ 1 mm bubbles, also emphasizing the huge size contrast between flapping droplets and jet drops (right); see (4), and in the same vein (5).

The reference scale for the bubble size (their radius *R*, see Fig. 1) is the capillary scale (we denote σ the liquid surface tension, *g* the intensity of gravity and *ρ*_1_ the density of the liquid)

(1) $$\begin{equation*}
a=\sqrt{2\sigma /\rho _1 g}={\cal O}(2\times 10^{-3}\rm {m}) \, \, \rm {in~water}.
\end{equation*}
$$

Jet drops (formed from small bubbles with *R* < *a* ) produce “giant” droplets (14) compared with films drops (issuing from large bubbles with *R* > *a*) that are not only finer, but also typically more numerous than jet drops (2,5,13).

**Fig. 2.**
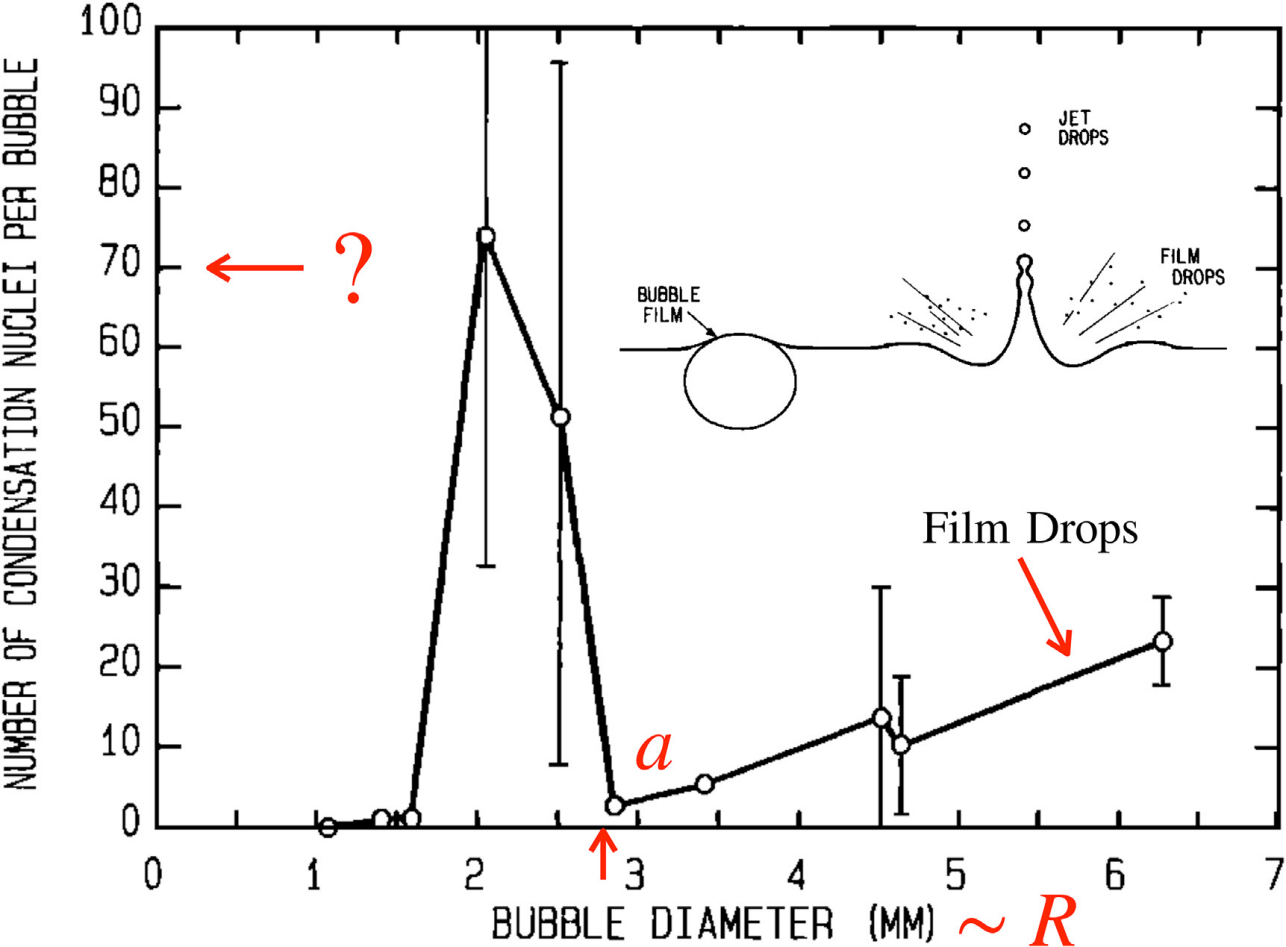
The “anomaly” discovered by Blanchard and Sysdek in the droplets production rate as a function of the bubble size: emission practically ceases for *R* ≈ *a*, in-between two regimes, one of them for *R* > *a* being characteristic of centrifuged film drops (section “Film and jet drops” ), the other for *R* ≲ *a* featuring a very large number of droplets per bubble being, at the time, more difficult to understand. Droplets from bubbles with *R* < 1mm were too small to be detected (adapted from Figs. 1 and 5 of (11)).

Blanchard and Sysdek (11), however, discovered an anomaly in the droplets production rate as a function of the bubble size (Fig. 2): emission practically ceases for *R* ≈ *a*, in-between two regimes, one of them for *R* > *a* being reasonably attributed to film drops, the other for *R* ≲ *a* being more difficult to understand, if not to admit; the reality of these observations has been challenged, criticized (15,16), before being confirmed (17), and tentatively explained (18). The phenomenon discovered by Blanchard and Sysdek was indeed, by their own consent, an anomaly because the corresponding droplets were at the same time too numerous, too small, and emanating from too large bubbles to be interpreted as jet drops.

### A new mechanistic understanding for the origin of submicron drops

An observation, not only solving the above mentioned paradox, but also shedding a new light on the nature of the relation between the ocean superficial activity and its contacting atmosphere was made recently (4).

There was never a question that film breakup lead to submicron aerosol (17,19–21), but the philosophy underlying all previous experimental studies and their interpretation was that the presence of an atmosphere in contact with the liquid pool is incidental: It was believed that the jet rising from a collapsing liquid cavity expands in a near-vacuum (jet drops), as well as the ligaments torn-off from the destabilizing rim travelling along the curved bubble cap (which produce the so-called film drops), and this belief had all the appearances of truth since the predictions based on that assumption were indeed found to be even quantitatively correct.

The possible role of the gaseous atmosphere may play in the production of bubble aerosols has never been questioned. Admittedly, the large density ratio between the standard atmosphere (density *ρ*_2_) and that of the liquid (water, density *ρ*_1_), of the order of *ρ*_2_/*ρ*_1_ = 1.2 × 10^−3^ on Earth, does not encourage to think the gaseous environment as a relevant partner in the process involved in the problem.

It was nevertheless known that the coupling between a thin liquid film and the environment in which it moves may, provided the film is thin enough and moves fast enough, give rise to an original phenomenon: *soap*films burst like flapping flags, as noted in (22), thereby solving another old problem, that of the spontaneous destabilization of opening hole rims in soap films (a few microns thick). The film undergoes a shear instability with the light, but nevertheless inertial environment, conferring to it a flapping motion which triggers, via a series of subsequent instabilities, its breakup into fine droplets.

It is building on this fact that a series of experiments was devised to check if the environment could play a role in the destabilization of the very thin, and thus, very fragile cap of small bubbles as they burst (4). A marked effect of the atmosphere was observed, since using bubbles with *R* < *a*, it was found that

1. Droplets emission is essentially suppressed in a light gaseous environment (Helium).
2. The number of droplets is strongly augmented while their size is measurably decreased in a denser environment (SF_6_, five times denser than air).
3. A flapping motion of the bubble cap is indeed involved.

Thereby heavily suggesting that most of the aerosols coming from small (*R* < *a*) bubbles should be understood as resulting from a coupling with the environment and that the formula giving in-fine their typical size should mandatorily incorporate the density *ρ*_2_ of the gas phase.

This new physical mechanisms appears to solve Blanchard and Sysdek’s paradox (who, stuck at finding a reason for it stated (16) “...*nature is being very subtle in hiding it from us*”) for having played with the only ingredient of the problem which had remained overlooked.

We discuss below how these new facts articulate with previously known ones, aiming at quantifying their relative importance.

## Mechanisms, bounds, and relative importance

### Jet drops

In the inertial limit, i.e., as long as viscous effects do not come into play, and for bubbles sufficiently small for gravity to be unimportant (*R* < *a*), the only lengthscale of the problem is the bubble radius *R* itself. Consequently, both the radius *r* of the jet emanating from the cavity collapse and the droplets diameter *d* subsequent to its capillary breakup must be proportional to *R*. The corresponding scaling laws (but not the prefactors) are obtained as follows: the liquid volume in motion ∼*R*^3^ at cavity collapse feeds a jet of radius *r* and length ℓ at breakup such that *R*^3^ ∼ *r*^2^ℓ, and $\ell \sim u\, t(r)$ with *u* ∼ *R*/*t*(*R*) the jet velocity, $t(R)\sim \sqrt{\rho _1R^3/\sigma }$ and *t*(*r*) ∼ *t*(*R*)[*r*/*R*]^3/2^ being the capillary times based on *R* and *r*, respectively (25). We find *r* ∼ *R*, and therefore, from the Plateau instability of the jet (for which *d* ∼ *r*, see e.g., (25)), the size of the droplets *d* ∼ *R*, and their number *N*(*R*) ∼ *R*^3^/*d*^3^ a constant, independent of *R*. Blanchard (13) found that

(2) d ≈ 0.1 R (the 10% rule of thumb)

(3) $$\begin{eqnarray*}
\textrm {and}\, \, \, \, \mathit{ N}(\mathit{ R})\lesssim 10
.
\end{eqnarray*}
$$

The jet drop process cannot affect bubbles larger than *a* since these larger bubble cavities have a free-fall time $R/\sqrt{gR}$ smaller than *t*(*R*).

At the lower end of the bubble size spectrum, the above result holds until viscous damping alters the cavity collapse dynamics, this occurring obviously for very small bubbles, those for which *R*^2^/ν_1_ < *t*(*R*) or, said differently, for $Oh=\eta _1/\sqrt{\sigma \rho _1 R}$ larger than some critical value and/or, expressed in terms of bubble size, for *La* = *R*/*R*_ν_ larger than some critical value if $R_\nu =\rho _1\nu _1^2/\sigma$ is a viscous radius. In practice, these values are *Oh* ≈ 10^−2^ that is *La* = 10^3^, corresponding to a viscous scale *R*_ν_ ≈ 10^−2^μm =10^−8^m and a bubble radius of $R\approx 10\, \mu$m in water. Below that size, the cavity collapse is over-damped, and no jet is emitted (see e.g., (26), there are plenty of observations of this fact, including numerical).

**Fig. 3.**
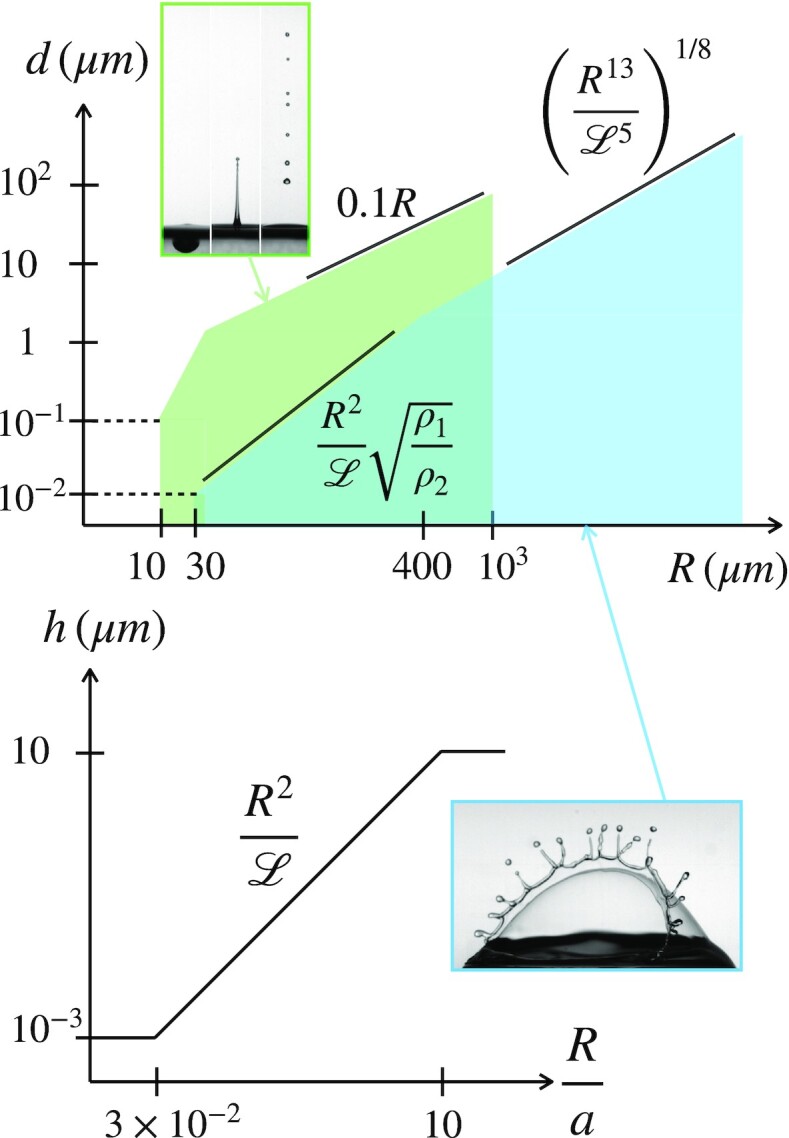
Top: sketch (original data in cited references) of the typical droplets size *d* dependence on the bubble radius *R* according to the different processes: the jet drop mechanism (green, *d* = 0.1*R* for *R* < *a* ≈ 10^3^μm, and ten time smaller for *R* around 10 μm (13)), the centrifuged film drops [blue for *R* > *a*, where *d* ∼ (*R*^3^*h*^5^)^1/8^ (2)], and the flapping film droplets [blue for 30 < *R*(μm) < 10^3^ where *d* depends explicitly on the ratio *ρ*_1_/*ρ*_2_ (4]). Bottom: The cap thickness at breakup *h* of a surface bubble as a function of its radius *R*. The relationship *h* ∼ *R*^2^ was first evidenced by Spiel (3), measured quantitatively in (2) who introduced the length ${\cal L}=\beta a$ with β ≈ 10^3^, a lengthscale whose nature was tentatively explained in (23), and measured independently in (24).

Interestingly, the jet is faster, and the droplets smaller than those expected from Eq. (2) in the close-to-critical region (namely in a region of radii around $R=10\, \mu$m spanning over a decade, see e.g., (27)). Droplets sizes can be as small as 0.01*R* giving, for *R* = 10 μm, a lower bound droplet size of the order of

(4) $$\begin{equation*}
d\gt 10^{-7}\textrm {m}=0.1\, \mu \textrm {m}
,
\end{equation*}
$$

formed by a jet drop process (Fig. 3).

This deviation from the “10% rule” in Eq. (2) was already known from Blanchard (13), and its interest in it has been revived by refined numerics (28) and experiments (29), followed by several other contributions of the like (see e.g., (30–33)). The argument is subtle: viscous damping delays cavity crunch, leaving time for capillary waves to travel along its wall, and to focus at its bottom, fostering a concentrated, fast jet. The ejected size *d* of the first droplet and its velocity *u* have been represented by complicated yet quantitative formulae in (34) and (35,36), expressing that *d* can be 10 times smaller and that *u* can be 10 times larger than expected from the 10% rule [namely, according to Eq. (2), *d* = 0.1*R* and *u*(*R*) ∼ *R*/*t*(*R*), respectively, see (28,33,37) and a review of these predictions in (8,31)].

It remains that there is no way, by this process, to form a droplet smaller than 10^−7^m, a size that, incidentally, is independent of the nature of the environing gas according to the above mentioned predictions.

### Film (centrifuged) drops

A bubble floating at the surface of a water pool will not stay there forever, but understanding its lifetime is anything but obvious. However, the droplet sizes depend directly on the bubble cap film thickness at burst *h*, which is orders of magnitude thinner than *R*. The same ingredient responsible for keeping a bubble alive (Marangoni stresses counter-balancing gravitational drainage of the film (2), a bubble with a clean cap does not exist), is responsible for its death (the nucleation of a hole on its cap (38)). It has been argued (2) that an isolated bubble lifetime is compatible with $t_b\sim t_v ({\cal L}/a)^{3/2} (R/a)^{1/2}$ with *t_v_* = η_1_*a*/σ a viscous time, and ${\cal L}\sim a Sc^{2/3}$ (with *Sc* = ν_1_/*D* the Schmidt number of the impurities driving the Marangoni stress, see (23)), a scenario that has received some experimental support (24), thus giving a status to the lengthscale ${\cal L}=\beta a$ (with β = *Sc*^2/3^ ≈ 10^3^ in practice) linking the thickness of a bubble cap *h* at burst to its radius *R*

(5) $$\begin{equation*}
\mathit{ h}=\frac{\mathit{ R}^2}{\cal L}
.
\end{equation*}
$$

The *h* ∼ *R*^2^ scaling was discovered by Spiel (3), the length ${\cal L}$ was introduced in (2).

A certitude, however, is that systematically, the lethal hole nucleates at the foot of the bulle cap (and we understand why (2,23)). The opening rim collecting the liquid constitutive of the cap thus travels, at the Taylor–Culick speed $v=\sqrt{2\sigma /\rho _1 h}$ along the curved cap (with radius of curvature *R*), over a maximal extent given by *E* ∼ *R*^2^/*a*. In the meantime, it develops a Rayleigh–Taylor instability triggered by the centripetal acceleration *v*^2^/*R*, and fragments, provided the residence time of the rim on the cap *T* is larger than the characteristic time of growth of the centrifuge instability *t_RT_*, which is as long as

(6) $$\begin{equation*}
T\gt t_{\rm RT}, \, {\rm with} \, \, \, T=\frac{E}{v},\, \, {\rm and}\, \, \, t_{\rm RT}\sim \sqrt{\frac{\rho _1}{\sigma }}\left(Rh\right)^{3/4}
.
\end{equation*}
$$

Bubbles with radii *R*/*a* > β^−1/3^ fulfill the above criterion and produce droplets with average diameter *d* and number *N*(*R*) given by (2)

(7) $$\begin{equation*}
d\sim \left(\frac{R^{13}}{{\cal {L}}^5}\right)^{1/8},\, \, \, {\rm and}\, \, \, N(R)\sim \beta ^{7/2}\left(\frac{R}{a}\right)^{9/8}
.
\end{equation*}
$$

**Fig. 4.**
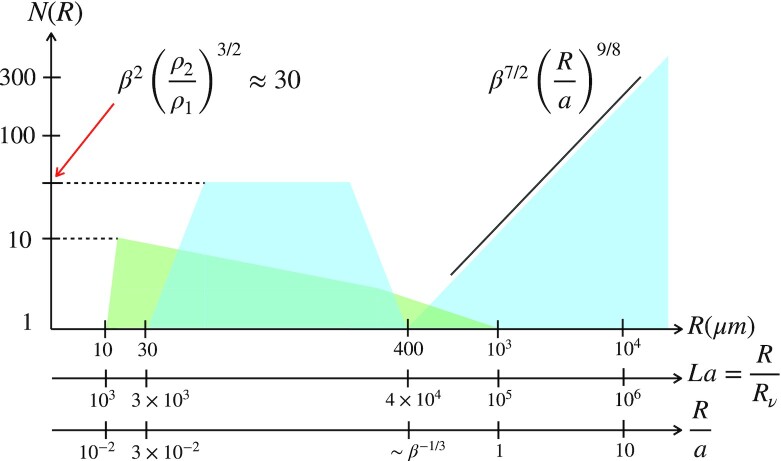
Schematic of the droplets production yield *N*(*R*) according to the bubble size *R* and domains of prevalence of the different mechanisms (see also Fig. 2). Centrifuge film drops (in blue) prevail for *R*/*a* > 1. The flapping drops emitted from bubbles with radii 30 < *R*(μm) < 400 are more numerous (in blue) than jet drops (in green), they also produce finer droplets, as seen from Fig. 3 (same color coding and references for original data; *R*_ν_ ≈ 10^−2^μm in water).

The number of droplets generated this way can reach hundreds per bubble, much more than through the jet drop process (for which this number is ten at most, see Fig. 4).

Bubbles smaller than a fraction of a millimeter ($a\beta ^{-1/3}\approx 0.4 \, \textrm {mm}$ or so) are insensitive to this mechanism, because their cap rim has no time to destabilize before ending its journey along the extent *E* (this criterion is consistent with the extinction in droplets emission found below about *a* in (11)), providing a minimum droplet size of the order of

(8) $$\begin{equation*}
d\gt 10^{-5}\textrm {m}=10\, \mu \textrm {m}
,
\end{equation*}
$$

formed by this centrifuged film process. It is, like the jet drop emission process, insensitive to the gas phase density.

### Flapping (film) drops

The fragmentation of millimetric bubbles and below (i.e., smaller than about *a*β^−1/3^) is due to the presence of a gas phase: it is an empirical fact that when this phase is made evanescent (i.e., ρ_2_ lowered appreciably, like replacing air with helium), droplets emission practically stops (4).

A coupling involving the gas as a driving force for the liquid fragmentation has thus to be sought, which might look unconventional, although familiar in different but related situations (39–41), but is mandatory.

A possible mechanism, known to operate in very similar instances, is the flapping, Squire instability of the moving film whose conditions of occurence, and main features, have been described in (22,40). Under these circumstances and for a rim receding at the Taylor–Culick speed *v* above, the characteristic time of growth of this instability *t*_S_ for an instability wavelength λ are such that (42)

(9) $$\begin{equation*}
t_{\rm S}\sim \sqrt{\frac{\rho _1}{\rho _2}}\frac{\sqrt{\lambda h}}{v}\sim \frac{\rho _1}{\rho _2}\frac{h}{v},\, \, \, {\rm and}\, \, \, \lambda \sim \frac{\sigma }{\rho _2 v^2}\sim h\, \frac{\rho _1}{\rho _2}
.
\end{equation*}
$$

As explained above for the centrifuged rim, this instability will have time to develop provided *t*_S_ < *T*, a condition, which constrains the environment only, independently of the bubble size *R* since it translates in

(10) $$\begin{equation*}
\beta \, \frac{\rho _2}{\rho _1}\gt 1
.
\end{equation*}
$$

This particular prediction is nicely consistent with the empirical observation showing that the case of air is just marginal (ρ_2_/ρ_1_ ≈ β^−1^), that very few droplets are emitted in helium, and that substantially more droplets are found in SF_6_ than in air. That alone is a very strong support for this mechanism, where the inertial, nonevanescent environment is, through shear, the driving force of liquid fragmentation.

The droplets typical size is given by

(11) $$\begin{eqnarray*}
d&\sim \sqrt{v\, t_{\rm S}h} =\sqrt{\frac{\sigma }{\rho _1\frac{v^2}{\lambda }}}
,
\end{eqnarray*}
$$

(12) $$\begin{eqnarray*}
&\sim h\sqrt{\frac{\rho _1}{\rho _2}}=\frac{R^2}{{\cal {L}}}\sqrt{\frac{\rho _1}{\rho _2}}
.
\end{eqnarray*}
$$

Droplets are expected to be smaller than for the standard centrifuge mechanism since the relevant acceleration *v*^2^/λ detaching the ligaments from the film here is larger than *v*^2^/*R* in the standard film drop process described before, that condition (λ < *R*) being largely fulfilled when *t*_S_ < *E*/*v* = *T*.

An upper bound of the number of fragments emitted with the size *d* above per bubble *N*(*d*|*R*), if all the liquid volume contained in the bubble cap ∼*E*^2^*h* is converted into droplets with the size given above, is expected to be independent of *R* since

(13) $$\begin{equation*}
N(d\vert R)\lesssim \frac{E^2 h}{d^3}\sim \beta ^2\left(\frac{\rho _2}{\rho _1}\right)^{3/2}\approx 30\, \, \, \textrm {in~air},
\end{equation*}
$$

an order of magnitude compatible with the early measurements in (11), and recent ones (4).

The droplets sizes are, according to this mechanism in Eq. (12), smaller in a denser environment, also consistent with the observations reported in (4). They present in addition an expected strong decay with the bubble size *R*.

What limits the droplets size in this process? The strong dependence of *d* on *R* reflects the dependence of *h* on *R* (the Spiel dependence *h* ∼ *R*^2^). To date, we have confident measurements of *h* down to $h(R\approx 1000\, \mu \textrm {m})\lesssim 0.1\mu \textrm {m}=10^{-7}\textrm {m}$ (2). But we have no experimental evidence that the same law will apply down to smaller thicknesses. There is no reason to think that *h* cannot go down to the thickness of Newton black films, limited by intermolecular forces (nanometers in thickness). It is possible to prepare nanometric films (43) and they can be, under strict conditions of water purity and ambient vapor saturation, long lived (44). Repulsive forces (the disjoining pressure) will probably slow-down the thinning process of these films as they approach such small thicknesses, but there is, to our knowledge, no serious reason to think that this would alter the Spiel dependence we have measured down to 10^−7^m. We also know that some surfactants hinder hole nucleation of the bubble cap, which then thins by evaporation down to dramatically small thicknesses at burst (45,46).

So, under this –pivotal yet unjustified– assumption, which we make in the absence of an alternative plausible scenario, and reliable measurements, a $R=1000\, \mu \textrm {m}$ bubble will have $h=1\, \mu \textrm {m}$ and will produce $d=30\, \mu \textrm {m}$ droplets, a $R=100\, \mu \textrm {m}$ bubble will have $h=0.01\, \mu \textrm {m}=10^{-8}\textrm {m}$ and will produce $d=0.3\, \mu \textrm {m}=300 \, \textrm {nm}$ droplets (like in (4), remember that the dried size of a 35 g/l salted water droplet is four times smaller than the original droplet). Extrapolating further speculatively to smaller bubbles, a $R=30\, \mu \textrm {m}$ bubble fitted with a rigid black film with $h=1\, \textrm {nm}=10^{-9}\textrm {m}$ would break into fragments of order

(14) $$\begin{equation*}
d=0.01\, \mu \textrm {m}=10^{-8} \textrm {m},
\end{equation*}
$$

in size, the smallest drop, which may be formed from a bursting bubble. This size coincides, and this is probably not a pure coincidence, with the size of the smallest aggregates attributed to the ocean surface activity (7). There are, however, multiple other sources of aerosols in this range of sizes and smaller (47).

**Fig. 5.**
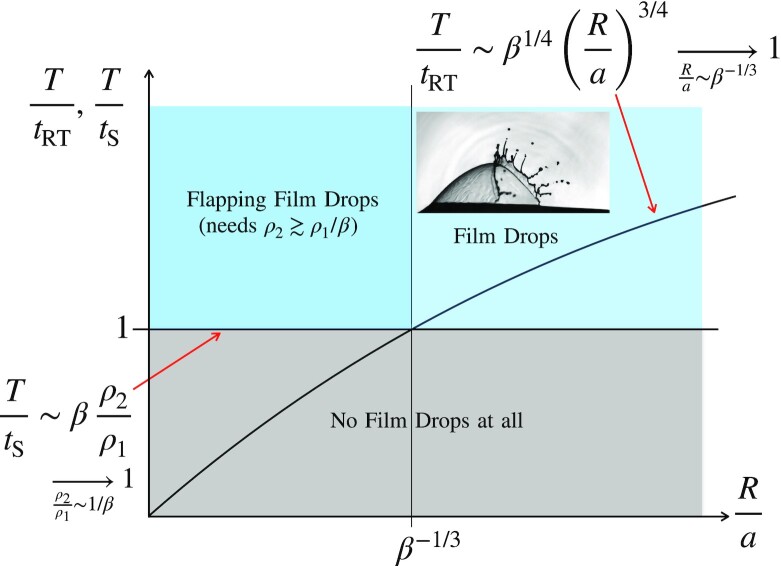
Synthetic diagram showing the required conditions, and domains of existence of the two film drops emission mechanisms (4). The regular, centrifuged film drops require that the rim residence time *T* ∼ *E*/*v* be larger than the Rayleigh–Taylor instability time of growth based on the centripetal acceleration *v*^2^/*R*, imposing that *R* > *a*β^−1/3^. Flapping film drops production require that the Squire instability growth time is smaller than *T*, which imposes that the gaseous medium is dense enough, i.e., *ρ*_2_ ≳ *ρ*_1_/β.

This new mechanism completes the overall picture of bubbles bursting at an interface in contact with a gas phase (see the diagram in Fig. 5).

#### Remarks for consistency

- Dealing with lengthscales appreciably smaller than a micron within a framework assuming the existence of a continuum needs, at least, to make sure that the relevant scales are larger than the mean free path of the gas molecules. It is roughly $10^{-7}\, \textrm {m}$ in air at atmospheric pressure while the minimum wavelength λ of the flapping instability (the one which requires a continuum) is ten times larger for *h* of the order of a nanometer. The continuum approximation is thus safe.
- We have neglected the development of a viscous boundary layer in the gas at the contact of the moving liquid film. The Squire (Kelvin–Helmholtz) instability will not be altered by a continuous transition of the velocity profile between the film (moving at *v*) and the gaseous medium (immobile) as long as the viscous boundary layer thickness $\sqrt{\nu _2 T}$, say, is much thinner than the wavelength λ = σ/*ρ*_2_*v*^2^ expected from the pure discontinuous idealization of the problem (42). This requires that the bubble size should be larger than
  (15) $$\begin{equation*}
R\gt \beta ^{3/2}\left(\frac{\rho _2}{\rho _1}\right)^2\frac{\nu _2}{\sqrt{a g}}\approx 3\, \mu \text{m},
\end{equation*}
$$
  a condition, which is amply satisfied since the minimal bubble size producing the smallest fragment in Eq. (14) is $R=30\, \mu \text{m}$.
- Of course, the reasonings here are for mean droplets sizes *d* ≡ 〈*d*〉. Equipped with the appropriate dispersion around the mean (which are well represented by Gamma distributions Γ_*n*_(*d*/〈*d*〉), each subclass of bubble size for each mechanism in each domain of prevalence, with their appropriate order *n* and weight measured by *N*(*d*|*R*) give rise to a “sea spray distribution” in the spirit of what is done in (2) and (8). Explicitly, if *q*(*R*) denotes the bubble radius distribution at the sea surface, and since the different mechanisms for droplets production are essentially independent of each other, the number histogram *n*(*d*) of the aerosols sizes *d* is obtained by simple summation as
  (16) $$\begin{equation*}
n(d)=\int q(R) N(\langle d\rangle \vert R)\Gamma _n(d/\langle d\rangle ){\rm d}R
.
\end{equation*}
$$
  This mechanistic approach can be compared to the extensive field measurements (8,48) and explains why, because each mechanism presents a dispersed droplets size spectrum around its preferred lengthscale, the “sea spray distribution” is nevertheless a continuous function of *d* although composed of droplets coming from very different sources.

One must keep in mind that none of the above listed drop formation process is fully efficient. Not all fragmented droplets finally produce aerosols: air drag exerted on jet micrometer-sized drops prevents them from rising appreciably high above the surface, before they settle back. The ascending motion of a droplet is all the more severely damped that it is small (within a time *d*^2^/(η_2_/ρ_1_), of the order of a fraction of a millisecond for a micronic droplet, see (13) pp. 112–115). How much a fraction of these drops can penetrate free air to contribute to the aerosol spray far from the surface is still an opened question. For example, the simulations in (31) show a bubble with *La* = 1000 (that is *R* = 60 μm) producing more than ten jet drops. This very large number is not backed by any experimental observation to date. In fact, experimental measurements (49) have shown that a bubble with *R* ≈ 60 μm produces of the order of only one jet drop per bubble on average, suggesting either that there are much less jet drops produced than (31) claims, or that most of them have been stopped by air drag. The latter option would suggest that an evanescent, drag-less environment would favor droplets production, while the experimental findings in (4) display a marked opposite trend: a denser environment fosters droplets production (an experimental fact) as it favors bubbles cap flapping (the model proposed by (4), supported by analogue experiments, see Section ‘Flapping film drops’).

Similarly, many film drops are ejected in a direction nearly parallel to the water surface plane, several rebound on it, and some of them coalesce with the pool. These phenomena and discrepancies are in need of clarification.

## Bubbles production conditions and role of the environment

For jet and film drops to be produced, the corresponding bubbles must reach the water/air interface. When they had a chance to do so, physico-chemical parameters like liquid viscosity, surface tension and density, water contamination, and temperature gradients influence (in addition to their radius *R*) the bubble lifetimes, and therefore the thickness of their cap at burst *h* and the corresponding aerosols outcome sizes *d*.

### Bubble sizes distribution entrained by breaking waves

The distribution of the bubble sizes *R* entrained by a breaking wave under the surface in the liquid bulk has received extensive scrutiny (50–54). The typical radius distribution *Q*(*R*) under a breaking wave is robust, and presents two power law-like regimes separated by the so-called Kolmogorov–Hinze lengthscale ℓ, of the order of 1 mm in the ocean (50). It is usually admitted that Kolmogorov’s turbulent scaling suits at determining the size ℓ of the largest stable bubble in a turbulent liquid, for which the inertial stress *ρ*_1_*u*(ℓ)^2^ equilibrates capillary confinement σ/ℓ, with $u(\ell )\sim (\epsilon \, \ell )^{1/3}$. The typical turbulence dissipation rate immediately after the breaking wave event is ϵ ≈ 0.1 − 1 m^2^s^−3^, while the typical turbulence dissipation rate under a breaking wave field is rather ϵ ≈ 0.001 − 0.01 m^2^s^−3^ (the broad interval is due to the transient character of the breaking event, as well as to the large variations in breaking strength in the field (55)) and we have (56)

(17) $$\begin{equation*}
\ell \sim \left(\frac{\sigma }{\rho _1}\right)^{3/5}\epsilon ^{-2/5}\approx {\cal O}(1\, \textrm {mm}).
\end{equation*}
$$

The above relation was found by Hinze (57) to fit convincingly well the maximum droplets sizes in a water/oil emulsion recorded earlier by Clay (58). Systematic size distribution of bubbles immediately entrained by breaking waves in the field are still lacking, with few direct observations due to the difficulty of measuring bubbles just below the water surface (50,59–61). These measurements qualitatively corroborate our knowledge based on laboratory experiments, and it is generally agreed that

(18) $$\begin{equation*}
Q(R\ll \ell )\sim R^{-3/2},\quad \textrm {and~}\quad Q(R\gg \ell )\sim R^{-10/3}
,
\end{equation*}
$$

are good fits, with various possible interpretations (10,50,62).

#### The cascade dogma and a variant

The “cascade” dogma has been inherited from the representation of spectra in turbulence: let for instance ∫*F*(*k*)d*k* be the variance of the concentration fluctuations of a passive pollutant carried by a turbulent flow with velocity increments *u*(*k*) ∼ (ϵ*k*^−1^)^1/3^ and *t*(*k*) ∼ *k*^−1^/*u*(*k*) a characteristic cascade transfer time associated with scale *k*^−1^. Until the diffusive dissipation scale is reached (see Batchelor (63)), the fraction of variance *F*(*k*)δ*k* = *F*(*k*^′^)δ*k*^′^ is conserved as *k* → *k*^′^ transits toward the diffusive cut-off. Therefore (δ*k*^′^/δ*k* = *k*^′^/*k*), at equilibrium, the variance flux

(19) $$\begin{equation*}
\frac{kF(k)}{t(k)}\, \, \textrm {is~constant,}
\end{equation*}
$$

leading to *F*(*k*) ∼ ϵ^−1/3^*k*^−5/3^ [and when *t*(*k*) is independent of *k*, then *F*(*k*) ∼ *k*^−1^, see (63)]. The same reasoning was applied (62) to the bubble fragments spectrum *Q*(*R*) from a conserved volume ∫*R*^3^*Q*(*R*)d*R*. As a bubble fragments into child bubbles, the corresponding volume fraction *R*^3^ × *Q*(*R*)δ*R* in the distribution around *R* is conserved, so that

(20) $$\begin{equation*}
\frac{R^4Q(R)}{t(R)}\, \, \textrm {is~expected~to~be~constant,}
\end{equation*}
$$

hence the *R*^−10/3^ dependence above, holding as long as *R* > ℓ.

**Fig. 6.**
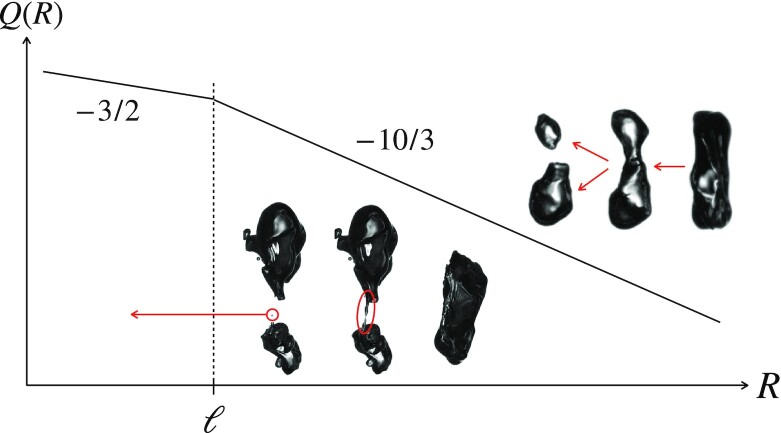
Sketch (original data in cited references) of the distribution of bubbles sizes *Q*(*R*) in a turbulent medium as interpreted within the “cascade” picture (62), leading to *Q*(*R*) ∼ *R*^−10/3^ for *R* > ℓ where ℓ is the Kolmogorov–Hinze scale in Eq. (17) and to *Q*(*R*) ∼ *R*^−3/2^ for those bubbles produced through the capillary instability short-circuit in Eq. (22) discovered by (10). Images adapted from (65).

Despite the fact that ℓ in Eq. (17) stands as a cut-off below, whose surface tension opposes to bubble deformation, smaller bubbles may be formed, now with the help of capillarity, which is the driving force for the destabilization of elongated, locally columnar-like bubbles, or ligament-like pieces of bubbles, whose role was first underlined by (10) in this context. These are stripped from large deformable bubbles with *R* > ℓ when they have the chance to adopt the idoine shape (see e.g., Fig. 6), and form faster as the ligament is thinner since now $t(R)\sim \sqrt{\rho _1 R^3/\sigma }$. Once formed with *R* < ℓ, these bubbles, which have “short-circuited” the cascade (see also (64) in the mixing context), are essentially stable. There is no cascade here, but a continuous feed of small bubbles from larger ones irrespective of their size, at the limiting rate *t*(*R*)^−1^ when the exploration frequency of the favorable bubble shape is large enough. We have (with *Q*(*u* > ℓ) ∼ *u*^−10/3^),

(21) $$\begin{equation*}
\partial _tQ(R)\delta R\sim \frac{1}{t(R)}\int _\ell ^\infty {\rm d}u\, Q(u)\delta p(R\vert u)
,
\end{equation*}
$$

where *p*(*R*|*u*) ∼ 1 × (*u*/*R*)^−1^ is the fraction of an elongated bubble of size *u* actually producing one (typically, at most a few) bubble of size *R*. Therefore, for *R* < ℓ,

(22) $$\begin{equation*}
\partial _tQ(R)\sim \frac{1}{t(R)}\int _\ell ^\infty {\rm d}u\, \frac{Q(u)}{u}\sim \ell ^{-10/3}R^{-3/2},
\end{equation*}
$$

consistent with the recent experiments in (10,65).

The cascade picture (which we know does not describe liquid atomization either, see Sections 9 and 10 in (42)), and notably its stationary character in Eq. (20) is highly questionable in wave breaking, admittedly the paradigm of a transient phenomenon, as well as the relevance of a reasoning relying on the unicity of ϵ which, we know is broadly distributed over three orders of magnitude, and therefore of ℓ (see in this respect the lucid remarks in (62,66)). At the opposite of the cascade picture, Garrett (62) even suggested that *Q*(*R*) could be a direct measure of the distribution of ϵ itself. These aspects certainly deserve further clarification.

### Rising bubbles actually reaching the surface

There is no observation of bubbles larger than a centimeter at the ocean surface. This large scale cut-off has probably the same origin as the one setting the largest raindrop, suggesting $R\lesssim a\sqrt{6}$ because larger, buoyant bubbles fragment as they reach the surface through a Rayleigh–Taylor instability (67).

Now, *Q*(*R*) is the source of the surface bubble radius distribution *q*(*R*) of which there is, to date, very few direct field observation (see nevertheless (68)), but these distributions have no reason to be identical. They are, in fact, not. One of the reasons is that very small bubbles (whose importance we have underlined regarding the jet drop mechanism) have no time to reach the surface before they dissolve in water: these bubbles, called small because they are not only stable viz turbulent stresses (*R* < ℓ), but also because their Stokes number *St* = *R*^2^/(ν_1_τ) is smaller then unity (τ is a stirring timescale of the flow, a fraction of the wave breaking time) behave essentially as a passive scalar and are trapped by the turbulent fluctuations in the liquid while they shrink by molecular dissolution (6,69). For instance, *R* ∼ 10 μm has *St* ∼ 0.1 and for *R* ∼ 100 μm, we have *St* ∼ 1. This means that bubbles smaller than ten microns have almost no chance of reaching the surface, while bubbles of order 100 microns will experience a reduced rise velocity due to turbulence (70,71) but should be able to reach the surface. The exact cut-off between these two extremes, presumably of order 50–100 μm, is uncertain. Small bubble entrainment by drop impact (Mesler entrainment (72)) together with the re-entrainment cascade of smaller bubbles consecutive of bubble bursting (73) are existing phenomenon which we do not expect to be statistically dominant.

Since the different aerosols formation mechanisms listed in Section “Perspective and facts” are sorted according to the bubble size, we stress that a detailed knowledge of *q*(*R*) is paramount for constructing a mechanistic sea spray generation function.This distribution must be the one of bubbles actually leading to droplets formation while we also know that bubbles in a raft at a pool surface coarsens in time. Bubbles merge with their neighbors without breaking, distorting their surface size distribution, which therefore depends on time, a phenomenon that has been actually witnessed in the ocean (68). Efforts should certainly be devoted in characterizing the (most likely transient) surface bubble population in open ocean conditions.

### Collective effects at the surface and role of water contamination

All the reasonings above are made at the scale of a unique, isolated bubble. Any ingredients reflecting possible collective phenomena are *de-facto* totally absent. However, when living at the surface, a bubble may interact with its neighbors, and collective effects might have to be accounted for; these depend on the water surface contamination and temperature.

While coalescence between adjacent bubbles just delays the phenomena seen for one bubble (2) without altering their nature, it does modify the surface size distribution *q*(*R*) when compared to the bulk one *Q*(*R*) by redistributing millimetric bubbles toward larger bubbles (68,74,75). Bubble coalescence is immediate in clean water, compared to contaminated water, where it is less efficient. As explained above, this alteration affects the relative importance of the different aerosols production mechanisms. However, it remains to be demonstrated that such strong variations in water quality exist in ocean conditions. Water temperature also leads to changes in the collective effects by delaying or accelerating the coalescence rate, hence modifying *q*(*R*). Next, the burst of a bubble may trigger the burst of nearby coalesced bubbles, there might be cascade phenomena of all sorts, but such processes have not been studied, nor even noticed to the best of our knowledge. The fundamental question remains: Regarding fragmentation, can an ensemble of bubbles be considered as the sum of its parts, or are there specific phenomena associated with a compact crowd?

### The role of sea temperature on aerosol production

The role of temperature on the total number of emitted sea spray aerosol is largely debated, if not uncertain (6,76,77), with recent field measurements showing an increase of the size and number of sea spray aerosol with temperature (78). Experiments with a plunging jet falling in water show an increase in surface bubble population with decreasing temperature leading to an increase in droplet population (76); while in other bubble production setups, the opposite trend was observed (21,77). However, no clear effect of temperature on air entrained is visible in field conditions measurements (6,48).

The different scalings presented in the Section “Perspective and facts”  lead to a sensitivity to temperature on the number and size of drops emitted by bursting, with each process affected in different ways, in particular through the changes in water viscosity with temperature. Liquid viscosity affects the focusing of capillary waves that leads to an ascending jet, resulting in larger and fewer drops when temperature increases. Film drop production is modulated by the effect of temperature through viscosity (moderately) and Marangoni stresses between the foot and the cap of the bubble (mostly, see (23)) in controlling the typical film thickness at bursting *h*, which itself controls the size and number of drops (with different scalings for centrifuge and flapping drops, see the Section “Perspective and facts”  ).

Bubbles are fragile objects extremely sensitive to minute changes in their environment, such as surface contamination or water temperature, for instance. While laboratory observations on jet drops tend to be robust and relatively insensitive to perturbations, a large statistical variability exists even there when considering film rupture: bubbles lifetimes are broadly distributed (2,23,24). This statistical variability makes the understanding of the role of temperature and contamination even more challenging and remains a source of uncertainties when trying to apply our fundamental understanding of bubble bursting to large scale models at the earth scale. Indeed, the parameterization of aerosols fluxes still suffers broad uncertainties (79), also because of our incomplete knowledge of the microscopic phenomena involved.

Finally, no clear trend on temperature effects on whitecap coverage, air entrainment and bubble size distribution in the field have been reported (6,48), to the best of our knowledge. Similarly, experimental work investigating air entrainment, foam decay, and bubble size distribution due to a falling jet has concluded that temperature effects are insignificant (80).

## Selective chemicals enrichment and transfer

A large fraction of aerosols coming from the sea surface activity span a size range from 10nm to 100 μm (see e.g., (81) and Fig. 3). Often called sea spray or sea salt aerosols, recent studies have nevertheless revealed that these aerosols comprise not only salt but also a significant proportion of organic species (82–85), with smaller droplets containing a larger mass fraction of organics. For instance, it is observed in (84) that particles smaller that about 0.1 μm have an organic mass fraction larger than 50%. It has also been known since Blanchard (86) that particules in seawater, such as microorganisms, bacteria, viruses (87,88), electric charges (89,90) and more recently microplastics, are enriched within smaller aerosols as compared to the liquid bulk (91,92). Aerosols are thus fundamental carriers of various substances from water to the air, thereby affecting a range of atmospheric phenomena and human activities.

This transfer process is however, selective and must be related to a specific drop production mechanism, but only a few studies have investigated the precise nature of the link. Jet drops sample water from below the bubble, and therefore contain more oxidized soluble organic species while lipids, polysaccharides, and some less hydrophilic proteins populate preferentially bubble caps and are thus mainly found in film drops (93). But not all film drops have the same composition since we also know that smaller droplets are more concentrated in organic chemicals. Nothing is know about the “distillation”—like process at play in the aging, and fragmentation of bubble caps, and its sensitivity to the bubble size.

The history of bubbles rise to the surface may also matter: it has been postulated that a bubble can scavenge particles like bacteria or viruses at its surface and concentrate them at its bottom as it rises. Those particles would thus be more enriched in jet drops than in centrifuged film drops (94); the status of flapping film drops in this respect is currently unknown.

Sea spray aerosols are efficient cloud condensation nuclei (95), an activity relatively insensitive to their organic content (96), and have an excellent ice nucleating ability—a vital aspect of cloud physics—an ability which is, on the contrary, largely controlled by its organic composition (97), and may have a biogenic origin (98). Both jet and film drops are thus likely to contribute to ice nucleation (49,99).

Aerosols are typically more acidic than the water from which they originate (100). One traditional explanation is that a small droplet will harvest, as it wanders in the atmosphere, more chemicals in its surroundings than a larger one, according to diffusion law (the concentration gradient at the droplet surface is inversely proportional to its size). But there is, in the light of the new findings regarding flapping bubble caps (4), an alternate interpretation: organics, insoluble surfactants, solid particles like plastics of all kinds are more concentrated at the water surface than in the bulk (45,101). This is also true for dissolved CO_2_ (which acidifies water) coming from the atmosphere. These impurities in the broad sense populate a thin layer at the liquid surface. It is precisely this layer, which is fragmented by flapping bursting bubbles (by contrast with jet drops, which come from deeper in the pool). In this view, there would be no post atomization enrichment, but rather a selective atomization of already enriched liquid, namely the one in the superficial layer of the pool. Floating plastic beads are indeed found to be enriched in fine aerosols (92), and may well be so by centrifugation of the surface layer in flapping drops.

This conjecture remains, as several others about the many mysteries mentioned above, to be investigated further.

It is precisely because air-sea exchanges are ruled by many intermingled, coupled phenomena and are mediated by very sensitive objects—bubbles—that precise experiments considering each effect in isolation are needed to reach certitudes. It is at this price that a framework to predict sea spray emissions, validated by field observations, will be reliably built from first principles.

## Data Availability

All data are included in the manuscript.
